# The potential of trace elements mapping in child’s natal tooth by laser ablation-ICPMS method

**DOI:** 10.1007/s40201-021-00611-2

**Published:** 2021-02-01

**Authors:** Aneta Olszewska, Anetta Hanć

**Affiliations:** 1 Department of Facial Malformation, Poznan University of Medical Sciences, Poznań, Poland; 2 Department of Trace Analysis, Faculty of Chemistry, Adam Mickiewicz University, Poznań, Poland

**Keywords:** Natal tooth, Trace elements, Imaging, LA-ICP-MS

## Abstract

**Purpose:**

Tooth enamel might provide past chronological metabolic, nutritional status and trace metal exposure during development. Thus, the trace elements distribution embedded in tooth tissues represents an archive of the environmental conditions. The choice of biomarker is estimated as critical to the measurement of metal exposure. Natal teeth are defined as teeth being present at birth.

**Methods:**

LA-ICP-MS provides a quantitative assessment of spatial distribution of trace elements in a natal tooth. The objective of the current study was to compare concentrations of building and other elements in a rare but reliable and valid biomarker - natal tooth.

**Results:**

It have been reported presence of potentially toxic elements: Pb, Cu, Mn, Cd, Ni distributed in prenatally and perinatally formed enamel and dentine.

**Conclusions:**

Analyses of deciduous enamel can provide answers into individuals’ earliest development, including critical pre- and perinatal period.

**Supplementary Information:**

The online version contains supplementary material available at 10.1007/s40201-021-00611-2.

## Introduction

The eruption of primary teeth typically begins at 6 months of age with incisor tooth in the mandible. However, in the chronology of tooth eruption it might be observed a severe alteration with presence of the first tooth at birth (natal teeth) or during the first month of life (neonatal teeth) [1]. Prematurely erupted primary teeth have also been described in the literature as congenital teeth, fetal teeth, predecidual teeth or dentition praecox. This rare condition has been the subject of curiosity and study since 59 B.C. by Titus Livius and others [2]. Nowadays these teeth also are the point of awareness of both parents and health professionals because of their abnormal clinical characteristic and concern of being swallowing or aspirated by the infant during nursing.

The prevalence of natal and neonatal teeth varies among different populations. In a study Zhu and King [3] the incidence of natal and neonatal teeth was ranging from 1:716 to 1:30,000, whereas Chow reported an incidence of 1:2000 to 1:3500 [4]. Prematurely erupted teeth more often are natal teeth, present at birth, compare to neonatal; it has been reported in an approximate ratio of 3:1 with a greater predilection to female infants [4]. According to the study Zhu and King the teeth affected the most often are the lower primary central incisors [3]. Bogendoff reported 85 % of natal teeth are mandibular incisors, 11 % maxillary incisors, 3 % mandibular canines and 1 % maxillary canine or molar. It has been observed eruption usually occurs in pairs and presence of more than 2 natal teeth is very rare [5]. In aetiology of natal teeth there have been consider many influencing factors: malnutrition, hypovitaminosis, osteoblastic activity in tooth germ related to remodeling phenomenon, hormonal stimulation, trauma, infection, febrile states, syphilis [4]. Even hereditary factors or an underlying syndrome can predispose to its occurrence. Hence, according to the current concept the presence of prematurely erupted teeth is attributed to superficial position of developing tooth germ, which predisposes the tooth to erupt early [6]. Mostly these teeth are precociously erupted from normal complement of primary teeth (99 %) and only 1 % of natal and neonatal are supernumerary teeth [7]. On the basis of anatomical, morphological and structural clinical characteristics natal teeth were classified into: mature - when they are fully developed in shape and comparable in morphology to the primary teeth; immature- when their structure and development are incomplete [4]. According to morphology natal teeth are described as normal or conical in shape, more yellow in color than normal, with poor or absent root development [8]. The color might be related to hypoplastic enamel, dentin or gingival covering. It has been reported natal teeth have tendency to discolor [7].

Histological investigations of natal teeth have demonstrated that the crown mostly is covered with thin hypoplastic enamel with varying degrees of severity, absence of root formation, ample and vascularized pulp, irregular dentin formation, lack of cementum formation and Hertwig’s sheath [6]. The concerned great mobility of natal teeth is related to incomplete root formation. It has been suggested that increased mobility may cause histological changes in cervical dentin and cementum thus resulting in degeneration of Hertwig’s sheath, decreased root formation and tooth stabilization [7]. Early eruption of natal, neonatal tooth is associated with hypomineralization of enamel which despite a normal ultrastructure, is reduced in thickness, dysplastic and covering only the two thirds of the crown. The incisal edge might lack enamel partially. The neonatal line which demarcate the pre- and postnatal regions is more readily visible in enamel in the upper part of the crown than in dentine [9]. The incremental lines in enamel correspond closely to those in dentine, thus allow to distinguish pre- and postnatal regions of dentine on the basis of neonatal line visualized in enamel [8]. The development of the primary teeth starts prenatally in 6 weeks in utero with formation of the first structures of primitive oral cavity (stomodeum). The dental tissue, enamel, starts to form during the third month of fetal development and proceeds in a highly-controlled, regular and well-defined pattern [10]. Earlier research based on this fact discussed hypothesis that the spatial distribution of some trace elements along incremental zones in human primary teeth may be applicable to determine pre- and neonatal exposure [9, 11]. Several studies have shown that enamel covering the tooth crown at birth is mineralized only in 30 % and acquires a substantial deposition of minerals postnatally after the entire thickness of enamel is achieved [12]. It suggests that the inorganic constituents of prenatally deposited enamel may be partly acquired during the postnatal period. Studies on human teeth by Berkowitz et all. [12] have shown that according to the fact that dentine is almost completely mineralized immediately after matrix deposition, the trace elements levels in pre- and neonatally formed dentin might reflect exposure during specific period of development. During amelogenesis in utero all primary teeth mineralize but complete enamel formation is observed within the first year of life [13]. It has been observed a characteristic pattern in enamel formation, because it begins at the cusps of the crown in waves that stretch down towards the tooth root. The cycle of enamel growth results in a daily layer called a cross-striation. The cross-striation forms by different rates of enamel deposition during a daily 24 hour period. These layers are formed appositionally and are separated by more clearly defined microscopic structures called striae of Retzius. Retzius lines are regularly spaced disturbances in the enamel that represent on average a week of enamel formation. Enamel mineralization is completed after the tooth erupts into the oral cavity by mineral ions from saliva [13]. The enamel surface of the outermost layers of 10 *µm* is of a slightly different histological structure. Enamel is the hardest tissue in the human body, composed of 90 vol% mineral, 4 % protein, and 6 % water [12]. The enamel comprises calcium, phosphate, water of a form known as hydroxyapatite (Ca_10_(PO_4_)_6_(OH)_2_). Developing enamel is composed of up to 18 % calcium and heterogeneous proteins [13]. The process of enamel mineralization is expected to proceed by interactions of amorphous calcium phosphate and a limited number of enamel matrix proteins ,which form about two thirds of the forming enamel volume [14].

The majority of the developing enamel matrix consists of amelogenin, which is protein-rich. The remainder of the enamel protein is a high-molecular-weight phosphoprotein, known as enamelin [14]. As the enamel continues to develop, the amelogenin is replaced as the enamel calcifies and then matures. During this process, over 90 % of the amelogenin is removed. If this process does not occur properly, e.g., during some metabolic disorders, enamel hypoplasia occurs. At a later stage, if calcification does not proceed properly during maturation, hypocalcification results. Mature enamel is an acellular calcified tissue with the highest mineral level [15]. Although the main mineral of enamel is a hydroxyapatite, natural tooth enamel always contains many other inorganic anions and cations (e.g. HCO_3_^−^, F^−^, CO_3_^2−^, SO_4_^2−^, Na^+^, Mg^2+^, K^+^, Cl^−^) and form a relatively complex apatite structure [16]. Apatite crystals in bones and teeth are far from being stoichiometric [15]. Instead, they are rich in defects and usually calcium-deficient [16]. To maintain electron neutrality upon calcium depletion, phosphate groups are protonated (HPO_4_^2−^) and/or phosphate groups are replaced with CO_3_^2-^ [17]. The crystallinity of enamel varies, depending on its purity and the degree of incorporation of other elements [18]. However, enamel is never a pure substance and always includes other inorganic or organic components during the developmental stage.

It has been demonstrated a strong positive correlation between the duration of the calcification period and the crystallite sizes/volumes and the negative correlation with the microstrain. Hence it might suggest possible causal relations among these variables, i.e., the longer the enamel calcification, the larger volume and the less lattice imperfections of elementary crystallites [19].

Enamel is laid down during development by specialized cells, and if a higher concentration of ions is available in the bloodstream, this trace element becomes incorporated by the ameloblasts in the developing enamel [15].

Ionic substitution is a common phenomenon in the oral cavity after tooth eruption. Enamel is an unique tissue ; being acellular once formed cannot be biologically repaired or replaced [13]. Thus throughout the life of a tooth, in the oral cavity, there are enamel demineralization/remineralization cycles that dictate the extent of mineral balance and tissue integrity or degradation. Human saliva has a buffering role and acts as a carrier of essential ions such as calcium, phosphate and fluoride, that can bring a change in the structure of enamel, promoting remineralization. The carbonate ion can replace hydroxyl or phosphate ions, magnesium can replace calcium, and fluoride can replace hydroxyl ions in the crystal lattice. These ionic substitutions have a significant influence on the behavior of apatite including its solubility [16].

Evaluation of mineral content of enamel might be a representative of environmental exposition to different trace elements during period of tissue formation as well as maturation [19].

Various analytical methods have already been tested for the elements analysis in teeth. One of them, LA-ICP-MS method is becoming increasingly utilized for analyzing tooth tissues in toxicological studies [19–21]. It is linked to ICP-MS capabilities such as large linear response, low limits of detection (µg/mg), and multi elemental analysis [21]. In addition, when ICP-MS is connected with LA, it may be used for elemental imaging in analyzed samples [22, 23]. Application of LA-ICP-MS for analysis of spatial distribution of elements in teeth, may be helpful in to monitored exposition on toxic metals and further incorporation in the body [24].

The aim of these study was to determine the content and distribution of the trace elements in the natal tooth using LA-ICP-MS technique. Mapping of the trace elements in the enamel and dentine of natal tooth, due to their incremental structure of growth, may indicate the chemical variability (exposure of the baby and mother to the toxic elements) during pregnancy.

## Materials and methods

### Sample and sample preparation

The natal tooth, mandibular central incisor, examined in this study was collected from a 14 days old child. The pregnancy was without any complication, the child was born full term with 10 Apgar score, with no complications during pregnancy or delivery. From medical history, mother was a heavy cigarettes smoker before pregnancy but she stopped in a second trimester. No other medication or diseases were recorded.

The crown of natal tooth was small in size, whitish in color and opaque. There was a mobility of this tooth observed and difficulty in breastfeeding. According to great risk of aspiration of this tooth a decision to extract it was made. The procedure has been postponed when a child is 10 days or more to avoid a great risk of potential hemorrhage and to allow a normal flora of the intestine to produce vitamin K. Shortly after extraction the tooth was cleaned with distilled deionized (DDI) water, brushed with soft toothbrush. In order to dissolve any remaining soft tissues the tooth was place in a plastic micro beaker with 2 % (w/v) papain solution. The tooth was washed several times with distilled deionized water and dried overnight (85 °C) in an oven.

The dried tooth was longitudinally sectioned with a diamond blade. During sectioning distilled deionized water was used to minimize heating and contamination due to sectioning. Half-tooth sections were washed with DDI and allowed to dry overnight. The tooth samples prepared in this way are stored in a polyethylene box for time analysis.

**Fig. 1:**
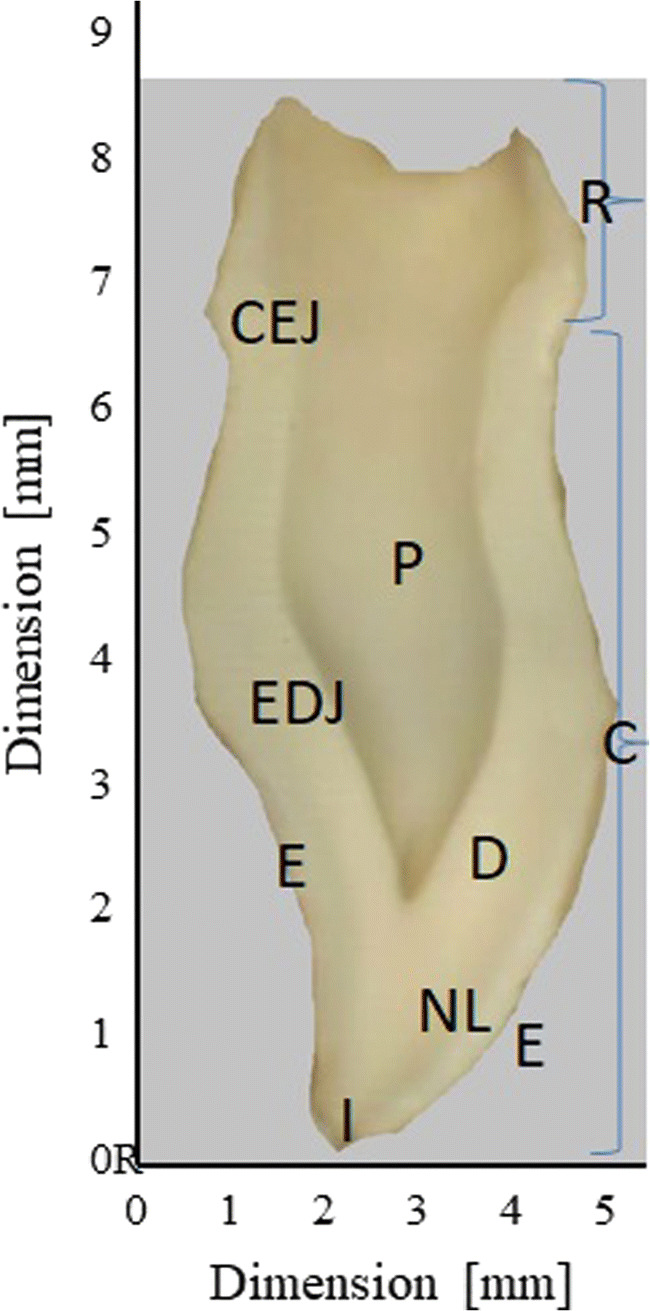
Diagram of cross section of a natal tooth: E-enamel, D-dentine, P-pulp, I-incisal edge, NL- neonatal line, C-crown, R-root, EDJ-enamo-dentinal junction, CEJ-cementoenamel junction (cervix)

The natal tooth exhibited decreased enamel and dentine thickness and had bigger pulpal space volume than the normal mandibular primary incisor tooth (Fig. 1).

### Instrumentation

Experiments were carried out using laser ablation system (LSX-500, CETAC Technologies, Omaha, NE, USA) working at a wavelength of 266 nm and hyphenated to a quadrupole ICP-MS instrument (Elan DRC II, PerkinElmer Sciex, Toronto, Canada). The ICP-MS torch was equipped with a quartz injector pipe with an inner diameter of 3.5 mm and the interface consisted of sampler and skimmer cones based on platinum. The ion optics were adjusted to yield maximum sensitivity and balanced mass response while ablating NIST SRM 610 Trace elements in glass. The optimization of parameters such as nebulizer gas flow, ion lens voltage and power of the plasma generator was carried out manually while monitoring ^24^Mg^+^, ^115^In^+^, ^232^Th^+^, ^238^U^+^ and ^232^Th^16^O^+^ ion signal intensities. Polyatomic oxide interference was evaluated and minimized by monitoring the ^232^Th^16^O^+^/^232^Th^+^ ratio and doubly charged ions ^42^Ca^2+^/^42^Ca^+^. Typical oxide formation and doubly charged anions were consistently under 0.2 %.

Imaging measurements were performed base line by line and always from left to right with 3 s delay at the end each line. During laser ablation was using a spot diameter of 50 and 10 nm distance between adjacent lines in order to reduce the risk of cross-contamination. Multi-elemental analysis by LA-ICP-MS was carried out using the analytical procedure described and developed on the basis of previous studies by Hanc et al. 2012 [24]. The quantification approach consisted of external calibration using matrix-matched calibration standards and ensured that samples and standards were similar in terms of chemical. The homogenate tooth powder was divided into fifth aliquots. Four of them were spiked with concentration of the preferred standard mix of standard elements solutions; the fifth samples was used as blank. Solid standards with following analyte content in µg g-1 were prepared: Ca, Mg, Sr (5, 50, 100, 500); Cd, Cu, Ni, Mn, Pb, Zn (1, 5, 20, 50, 100). In all measurement ^13^C^+^ ion intensity and ^31^P^+^ was monitored as internal standard of mass spectrometric measurements. Operating conditions for the optimized LA-ICP-MS system are given in Table S1.

After laser ablation analyses the natal tooth was mineralized in the microwave assisted high pressure digestion system. The natal tooth was accurately weighted and put into the quartz tube with 0.1 mL 65 % HNO_3_ (Suprapure Merck) and 0.05 mL 30 % H_2_O_2_ (TraceSelect Fluka) next placed in closed Teflon vessels into Microwave Digestion System (EthosOne, Millestone, Italy). The heating program was performed in two steps: (1) ramp time 15 min; (2) hold time 30 min; maximal power 1100W, maximal temperature 180 °C. The mineralized samples were quantitatively transferred into 5 mL volumetric vessels and filled with demineralized water. The digested solutions were analyzed by ICP-MS (external calibration) for determination of the multielemental concentration (see Table 1). To evaluate the elements concentration in the prepared matrix-matched standards, a part of each standards were digested too.

**Table 1:** Elements concentration [µg/g] of the natal tooth obtained by the solution nebulization ICP-MS

Content of elements in natal tooth determined by SN-ICP-MS [µg/g]	
Ca	249 ± 13
Cd	0.0545 ± 0.0032
Cu	0.432 ± 0.019
Mg	13 ± 1
Mn	1.84 ± 0.15
Ni	0.692 ± 0.031
Pb	0.284 ± 0.016
Sr	32.48 ± 0.31
Zn	76.4 ± 0.8

Elements concentration [µg/g] of the natal tooth obtained by the solution nebulization ICP-MS (the mean values ± SD; n = 3).

### Quality control

SRM NIST 1400 (www.nist.gov) and the standard addition method were used for quality control of the LA-ICP-MS results during analysis of teeth samples. Standard reference material was pressed into a pellet the same as matrix-matched standards. The calibration curves for determined elements were linear in the range of calibration standards. Coefficient of correlation (R) values were greater than 0.99 for all analytes. Precision values was calculated as coefficient of variation (CV) (%) ranged from 2.5–7.5 %. The trueness of the analytical results was expressed as recovery (%). Recovery values varied from 92–110 %. Limit of detection values were estimated as three times the standard deviation (SD) for the gas blank measured before the laser action began. The estimated detection parameters are as follows: 10µgg^− 1^ for Ca, 0.06µgg^− 1^ for Cd, 0.08µgg^− 1^ for Cu; 3µgg^− 1^ for Mg; 0.1µgg^− 1^ for Mn; 0.25µgg^− 1^ for Ni; 0.08µgg^− 1^ for Pb; 0.5µgg^− 1^ for Sr; 0.25µgg^− 1^ for Zn.

## Results

A total of 9 trace elements (Ca, Cd, Cu, Mg, Mn, Ni, Pb, Sr and Zn) were detected in the natal tooth sample by ICP-MS and LA-ICP-MS method. It was found the concentration of the different trace elements varied according to tissue (enamel, dentin) and also depends on the tooth region (incisal edge of the crown, developing root). The presence of potentially poisonous trace elements has been recorded in a sample of natal tooth. The trace elements are distributed heterogeneously in the dental tissues (enamel, dentin, pulp) varied according also to the tooth region (the crown, the root) [21]. Each element has its own characteristic distribution throughout the natal tooth. The use of laser ablation method allowed to study the spatial distribution of both building materials such as Ca and P and the potentially toxic ones (Pb, Cd, Ni) in the natal tooth. A photograph (Fig. 1) of a cross-section of a natal tooth showing the central pulp chamber and root canal and the layers of enamel and dentine where evaluation of distribution of trace elements has been done. Ca exist naturally in the sample matrix, as a main constituent of enamel and dentin, building a mineral part of it – hydroxyapatite [16]. Nevertheless, in a fully developed deciduous tooth Ca is as far as evenly distributed in enamel and dentine. Thus, Ca can serve as a baseline against which fluctuations in trace element intensities can be corrected [25]. In a case of natal tooth it has been noticed uneven distribution of Ca. The highest intensity of signal for Ca, was observed at the top of the crown, at the incisal edge. This is a region where mineralization of organic matrix starts [15]. Ca signal declined in dentine adjacent to the pulp chamber. The lowest intensity of signal for Ca is noted at the pulp region and in the area of developing root. This observed pattern of Ca distribution in a natal tooth might be influenced by intensive growth and development of dental tissues. It is known, teeth start to form in utero, then record birth as the characteristic neonatal line, and then continuously manifest daily growth lines in a dental tissues structure. However, after tooth erupts into the oral cavity it needs 2–2,5 years since the full maturation and mineralization of the dental tissues is achieved [15]. It was reported that Ca exhibits a chemical behavior similar to that of Sr, Ba and Mg [25].

**Fig. 2:**
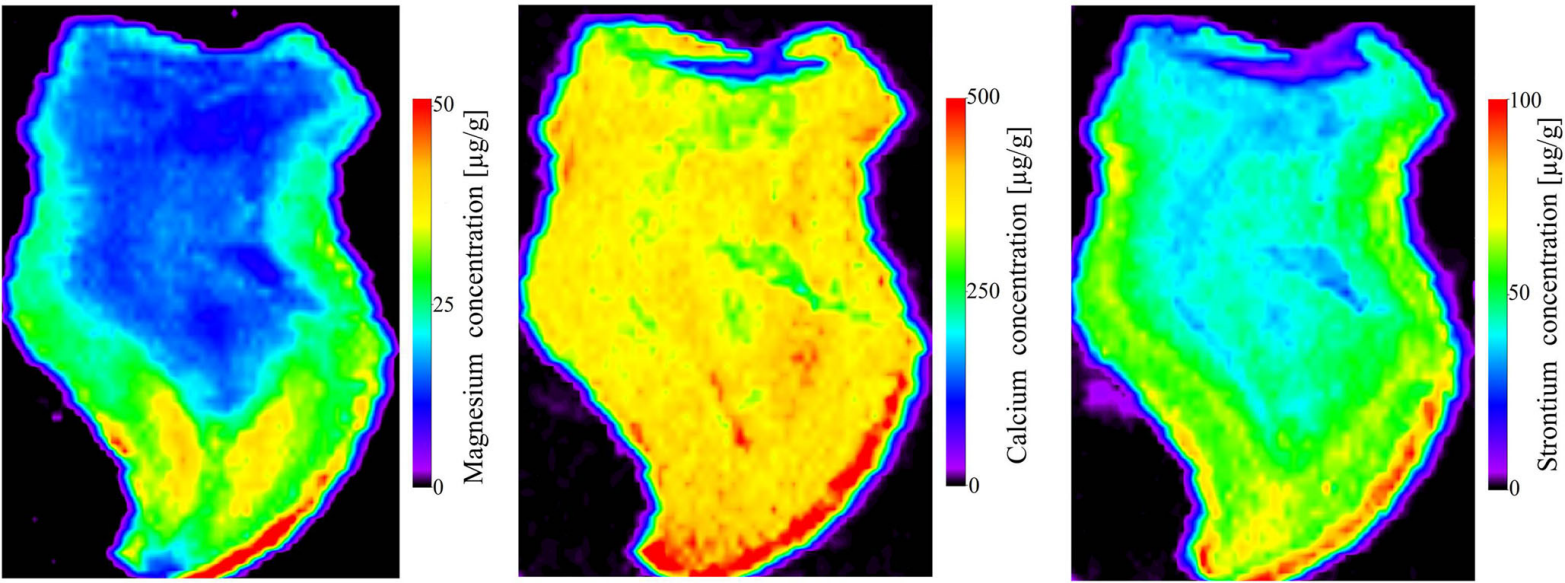
Distribution of ^26^Mg, ^43^Ca and ^88^Sr in cross section of natal teeth derived from laser ablation ICP-MS analysis

It has been observed a similar distribution of Sr to showed above that of Ca. Sr is not a building element, but due to its chemical similarity to Ca it shows similar distribution in the tooth structure. Higher signal intensity of Sr was observed at enamel-dentin junction with some spots of the highest intensity at the incisal edge/the top of the crown. Sr signal in enamel was higher than in dentine. Sr signal declined in dentine adjacent to the pulp chamber. The lowest value has been observed in the root region (Fig. 2). Dental enamel is approximately 97 % inorganic. Mature dentine is also highly calcified (70 %) and nearly inert, although less so than enamel. Enamel and dentine are predominately composed of hydroxyapatite crystals. Bioapatite crystal formation is under direct physiological control of the organism, but also influenced by environment conditions. Thus, besides building elements some other trace elements might be embedded into mineral structure of the hard tissues of the developing tooth.

**Fig. 3:**
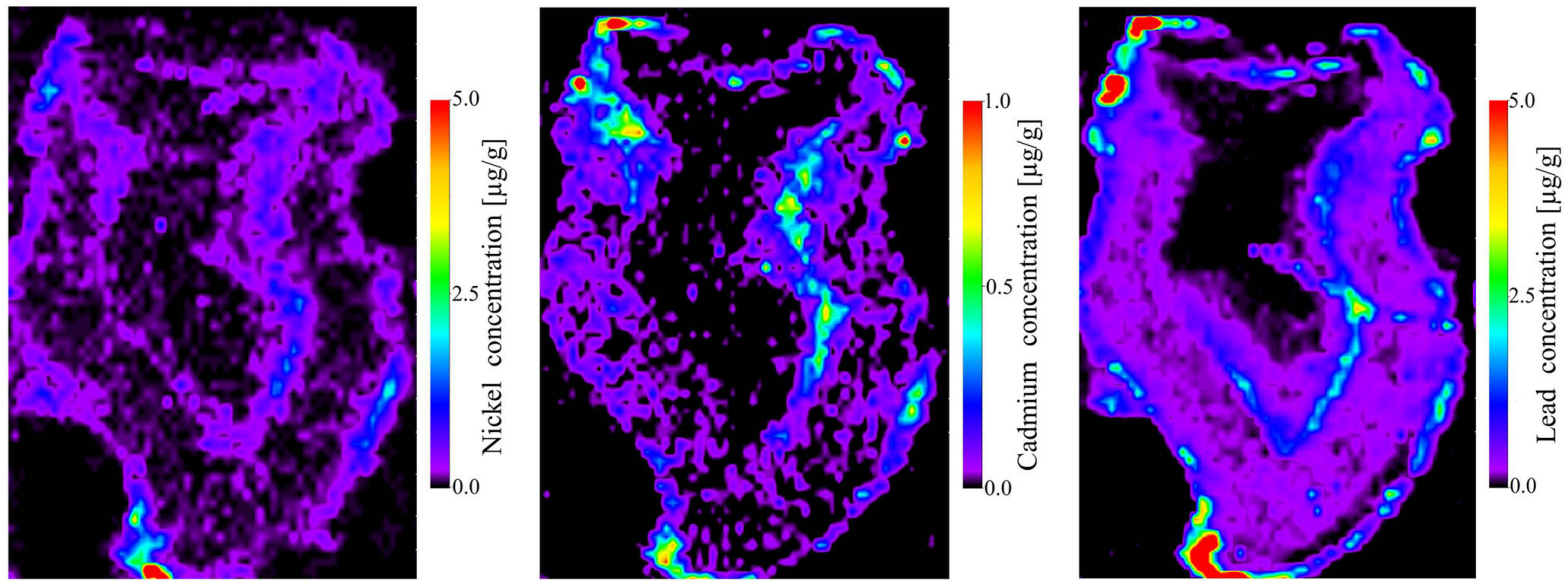
Elemental images of ^63^Ni, ^111^Cd,^208^Pb in natal tooth. Quantitative information is based on a matrix-matched laboratory standards

Mapping of the natal tooth could provide elemental information about distribution of physiological elements of dental tissues such as: Ca, P, Sr. A Fig. 3 showed distribution of main building elements in natal tooth (Fig. 3). In a recent study, the presence of potentially poisonous trace elements Pb, Cd, Ni, Mn, Cu was obtained in a sample of natal tooth. It is believed that the toxic effects of the trace elements are the most prominent during the pre- and neonatal periods, affecting the sensitive processes of a child development.

Lead, cadmium and nickel belong to highly toxic elements, especially for developing organism of the child [26, 27].

Lead is a toxin known to affect behavioral development and it easily crosses the placental barrier and accumulates in calcified tissue such as teeth [28]. Elevated intensities Pb were observed close to the enamel surface at the enamel rod and in the developing root area. Region of neonatal line shows low intensity lead distribution with intense spots along the neonatal line closer to the top and outer enamel surface. Lead in region of the dentine-enamel junction is evenly distributed and relatively low with the exception of a single spot. A zone of elevated signal intensity of lead can be seen along the dentin-enamel junction and extending toward the surface enamel. A homogenously distributed zone with a few intense isolated spots of lead can be seen in region dentin-enamel junction and neonatal line. Increase of lead signal intensity was observed at the bottom of the rectangle region of dentin-enamel junction. Region pulp-dentin shows an increased level of lead in the dentine compared to surface enamel. Elevated levels of Pb are often present in the dentine tissue. Region pulp-dentin illustrates the most intense zone of widespread lead present in the pulp tissue. High lead concentrations at the pulp might be because of the presence of residual organic matter in the pulp [29]. It have been reported in the previous study [27, 28]. Pb ions, at low concentrations, can replace Ca ions in HA crystals. The substitution decreases the size of enamel apatite crystals [15].

The highest intensity of signal for Cd was observed, similarly to Pb, as a few spots in the outer enamel at the incisal edge of the crown, at the dentin-pulp region and in the developing root region where vessels enter the pulp chamber.

The highest signal of intensity for Ni was observed in the outer enamel at the incisal edge and in the dentin-pulp region close to the cervical area (developing root). The intensity of signal for Ni elevated in the region pulp-dentin junctions. Nickel is incorporated in HA through substitution of Ca [30]. It bonds with O resulting in Ni_3_PO_4_ formation [31]. Accordingly with addition of Ni decrease of crystal domain site in tooth enamel was observed. Ghadimi et al. found a strong positive association with the presence of carbonate type B and the substitution of Ni in tooth enamel [32].

**Fig. 4:**
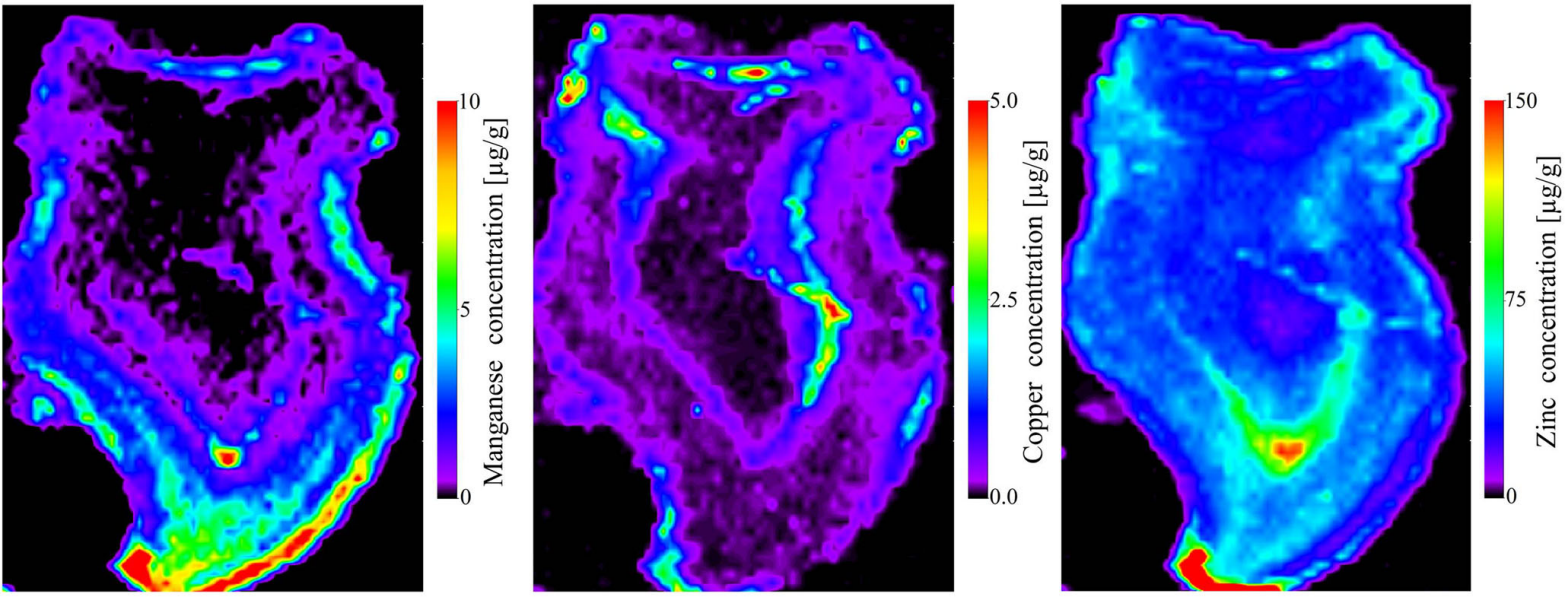
Elements ^55^Mn, ^65^Cu and ^66^Zn distribution observed in cross section of natal teeth

The results of these studies have shown the presence of Cu, Mn and Zn (Fig. 4). These are potentially dangerous for a child development if their concentrations exceed physiological limits. Distribution of Cu signal showed the highest level in the pulp region and in developing root area. It might corresponds with some impurities ,organic remnants in the pulp chamber. Some spots of higher intensity were observed at the top of the crown/incisal edge.

In region enamel surface high intensity of Mn is seen. Within the enamel, the highest Mn levels were seen at the outer edge. The inner enamel have lower signal of intensity for Mn. An interesting feature was the clearly higher Mn signal in the region of dentine near the incisal tip of tooth, which corresponds to the prenatally formed dentine in incisors. It has been demonstrated Mn:Ca signals in dentine bounded by the neonatal line were higher than in dentine formed after birth [30]. Region dentine-enamel junction shows an intense zone of Mn. High intensity of Mn is seen at either side of dentine-enamel junction. Pulp-dentin region has elevated zone of Mn at the bottom, close to the pulp. The highest intensity of Mn were observed at the top of the crown and the bottom of pulp region. Previous studies have shown Mn replaces Ca in hydroxyapatite although it does not change significantly the crystal size [31]. The distribution of zinc (Fig. 4) shows an elevated zone of Zn in pulp tissue region (at the top of the pulp chamber) and in dentine close to it. Uniformly distributed and high zinc intensities are at the incisal edge of the enamel and along the neonatal line. Compared to other metal ions with similar chemical properties, rather than being a toxic metal ion, zinc is an essential trace element. It has been demonstrated that the long-term, high-dose zinc supplementation may interfere with the uptake of copper. Thus, toxic effects of Zn are in fact caused by copper deficiency. Whereas excessive exposure to Zn is rare, zinc deficiency is widespread. It has a detrimental impact on growth, neuronal development, and immunity. Zinc deficiency caused by malnutrition and foods with low bioavailability is a far more common risk to human health than intoxication [32].

The findings of our mapping indicated that in the natal tooth trace elements were present mostly in outer part of enamel and in a dentine neighboring the pulp, reflecting prenatal exposure to heavy metals specifically during these very early periods of development.

## Discussion

Our LA-ICP-MS pilot study provides a qualitative and quantitative assessment of spatial distribution of trace elements in natal tooth. The objective of the current study was to compare concentrations of potentially toxic trace elements, in a rare but reliable and valid biomarker - natal tooth.

The low incidence of natal teeth in population which range 1:2000 to 1:3500 live birth [2], lead to great concern of researchers to this valuable and unique material for research. Decision of the natal tooth extraction can be justified based on a clinical indications such as increased mobility or anatomical and histological disturbances that if left may lead to local infection resulting in some cases in systemic complications [4]. Currently, the histological studies of natal teeth have reported differences compare to primary teeth: a lack of root formation, lack of Hertwig’s sheath and cementum formation, enlarged pulp chambers with an increased number of dilated blood vessels in the pulpal tissue and between the odontoblasts, irregular dentin and cell inclusions in the cervical region and interglobular dentine in the coronal regions [33].

Both the enamel and the dentin are mainly consisted of Ca, P, O and C, and the trace elements Mg, Sr, Al, Na and K [34]. It has been demonstrated in a study Zhao et al. 2002, that concentrations of trace elements varied according to a tissue. It has been demonstrated in our research that main building elements (Ca, Mg) were present mostly in newly formed enamel. While dentin contains more trace elements than enamel, mostly Mg, Na, K and Sr [21]. Incorporation of trace elements in both enamel and dentin is very limited and dependent on changing environmental conditions. Some impurities are under the level of their detection limit (such as As). The dentin HA and the enamel HA has different crystallinity [35]. The crystallinity of enamel HA is much higher than dentin also the HA in enamel is regularly arrange compare to dentine HA. The particles size of enamel HA in a fully formed tooth are more than twice bigger than in dentine in the same section [32, 33].

Several studies have shown that enamel covering the tooth crown at birth is mineralized only in 30 % and acquires a substantial deposition of minerals postnatally after the entire thickness of enamel is achieved. It suggests that the inorganic constituents of prenatally deposited enamel may be partly acquired during the postnatal period [36]. In our study it has been demonstrated distribution of trace elements varied according to the tissue and is higher in enamel especially in outer part and at the incisal edge, where mineralization starts. Studies on human teeth by Berkowitz et all. have shown that according to the fact that dentine is almost completely mineralized immediately after matrix deposition, the trace elements levels in pre- and neonatally formed dentin might reflect exposure during specific period of development [37]. The histological studies on dentine by Yoshiba et all 2002 [18] and Goracci et all 1999 [38] have shown that the inner part of dentine adjacent to the pulp blood vessels and occupied by odontoblasts would make it possible for a higher rate of exchange of trace elements between blood and adjacent dentine. The concentration of trace elements in inner dentine close to the pulp might be relatively higher than in outer dentine close to DEJ as previously has been suggested by Rabinowitz et all. (1993) [36]. Based on the study of Fergusson and Purchase 1987, lead levels are known to vary between different teeth types and also between primary and permanent dentition. It is therefore possible blood lead level measurements before six months of age would be more reliable than dentine, enamel lead level measurements [38]. Results presented by Arora et al. demonstrated that the lead distribution in dentine adjacent to DEJ in primary teeth may indicate the lead uptake during the pre- and neonatal periods. Thus improve the use of dentine-lead as a biomarker of lead exposure [38].

The results of the study Uryu et al. 2003, have shown the high level of Mn on the surface of enamel and lower levels in inner enamel similarly to the distribution of other metals such as Pb [39].LA-ICP-MS measurements have shown distribution of Pb in dentine, while a spot of analytical signal have been detected along the neonatal line closer to the top and outer enamel surface. This spot pattern may suggest temporary exposure of fetus to Pb, the content of Pb in natal tooth ranged 0.284 ± 0.018 µg/g. Earlier reports have also explained the higher concentration of trace elements on the surface of enamel. It might be a result of intensive deposition of ions during maturation phase of enamel mineralization. At this stage the outer enamel surface becomes hypermineralized compare to inner part of enamel [26]. The enamel surface is an important exchange zone for Ca, P and F ions with saliva [40]. Over the time it is observed at the surface of enamel accumulation of ions, involved in mineralization processes. Elevated elemental intensities at the enamel surface are potentially due to a process of re- and demineralization resulting from the interaction between saliva and teeth [25]. In a case of natal tooth trace elements distribution is influenced by blood circulation via placenta in prenatal life as well as postnatal exposure shown in corresponding layers of tissues. Higher Mn:Ca concentrations were seen in the region of dentine-enamel junction (DEJ), the Mn content in natal tooth was 1.84 ± 0.15 µg/g. In earlier studies Arora et al. have been reported higher metal concentrations in DEJ, this very complex structure [38]. This part of dentine is an outer dentine called mantle dentine. Berkovitz et al.2009 [41] have demonstrated profuse branching of dentinal tubules in mantle dentine. Some authors (Herr et al.) [27] have found the mineral composition of mantle dentin is different from other parts of dentine close to the pulp. The unique mineralization patterns of mantle dentine may appear as a zone of higher concentration level of some ions like Mn [38]. The other region where the higher concentration of Mn ions have been observed was the dentine adjacent to the pulp (dentine-pulp margin). The higher Mn levels in dentine close to the pulp may be a result of high density of blood vessels in this region. The intensive blood circulation facilitate the Mn ions exchange in the newly formed dentine and pre-dentine matrix [30]. An important region of tooth was zone of prenatally formed dentine at the incisal end of the crown (enamel rod). In our study a presence of highly demarcated high Mn zone was observed within the prenatally formed dentine.

The neonatal line is a histological feature in all primary teeth (and some first permanent molars) that demarcates the prenatally formed regions of teeth from those formed after birth [11, 36]. Thus, delineates prenatal and postnatal enamel in contrast to dentin that is constantly in exchange with the circulatory system. The developing fetus is supplied with trace elements from maternal sources via placenta. Depending on mother ion’s levels in blood some of them may be actively transported across placenta to a fetus [39].

Earlier reports of Ljung et al., have shown that some ions concentration (Mn) in maternal blood and breast milk are lower than in cord blood. It may explain the higher exposure to some metals (Mn) during the prenatal period than in early childhood [41]. The high concentration of Mn in prenatally formed dentine suggested the deciduous teeth may be a potentially useful biomarker Mn exposure during tooth development. The detailed map of the spatial distribution of ions in human primary teeth highlighted distinct patterns with key differences in enamel and dentine: very high Mn levels at the pulp-dentine junction and demarcated high Mn zone in prenatally formed dentine. This method may provide a chronological record of variation in elemental intensities in different regions of dental tissues that calcify at different times in life [42].

The trace element uptake of prenatal and early postnatal dental tissues draws upon the stores and overall nutritional status of the mother and the buffered environment of the placenta [36]. A breastfeeding infant continues benefits of maternal buffering but once weaning begins children are vulnerable to environmental differences impacting their nutrient uptake (e.g. socioeconomic status, disease, pollution or household size). Exposure of fetuses to foreign chemicals in utero is of serious public health concern. In general, the fetus is highly susceptible to the adverse effects of such chemicals and developmental deficit, congenital abnormalities and fetal mortality can result depending on the timing and strength of exposure. Some reports consider deciduous enamel to be a suitable medium for the assessment of in utero exposure to toxins [39]. The deciduous teeth exfoliate spontaneously thus provide samples for analysis that can be obtain non – invasively. Deciduous teeth are entirely unique biological tissues in that they permanently document prenatal as well as early postnatal environments. Teeth form incrementally at a known rate it is possible to identify specific developmental periods and assess changes in chemical composition along time. Deciduous teeth enamel in general forms from week 13 in utero up to 9 months postnatally thereafter essentially becoming inert. Formation of the enamel of the deciduous incisors starts about 3 months after conception and ends one month after birth [39]. Blood flow through the enamel is terminated after formation so the enamel records only the exposure during this formation period.

In a case of natal tooth a mother diseases, dietary habits, environment and lifestyle habits (cigarettes, drugs) are the most important factors influencing the trace elements concentration in dental tissues. The Cd presence in dental tissues might be influenced by mothers smoking habits that has been noted in medical history. In a case of natal tooth a mother diseases, dietary habits, environment and lifestyle habits (cigarettes, drugs) are the most important factors influencing the trace elements concentration in dental tissues. At the time of intensive growth and development children are vulnerable to the toxic effects of heavy metal exposure [42]. The most prominent period is the prenatal life. According to an immature blood-brain barrier and neuronal growth, migration and myelination, toxic exposure results in neurotoxic adverse consequences [41].

Prenatal and early environmental exposures have been implicated in developmental disturbances in children [41, 42]. The current study provides an examination of natal tooth and showed deposition of potentially toxic trace elements in dental tissues. Lead and mercury are widely studied neurotoxic elements, manganese have been implicated in the cause of neurodevelopmental disturbances has been demonstrated on mapping natal tooth. It is known teeth have been reported as reliable indicators of element exposure especially for lead. However, teeth have also been used as indicators of exposure to other heavy metals [43].

Further studies are needed to obtain more information about prenatal development and mineralization of teeth especially in the aspect of environmental exposure to pollution and toxic substances.

## Conclusions

LA-ICP-MS imaging has been showed in previous studies a powerful tool for mapping the spatial distribution of trace elements in teeth. Preliminary results indicate that clear demarcation of trace element deposition in regions of teeth associated with developmental periods were observed for trace elements Pb, Cu, Mn, Ni, Cd. Furthermore using a color spectrum mapping to determine the spatial distribution of trace elements in the pre-and postnatal dental tissues can be beneficial to improve the knowledge about qualitative aspects of natal teeth as biomarkers of exposure and the uptake of trace elements during tooth development and their effect on dental health and also for using teeth as trace element biomarkers in environmental biomonitoring studies.

## Supplementary Information

ESM 1 (DOCX 14.5 KB)
