# Supplementary material for: The potential of trace elements mapping in child’s natal tooth by laser ablation-ICPMS method
Source: J Environ Health Sci Eng. 2021 Feb 1;19(1):379–88. doi: 10.1007/s40201-021-00611-2 (PMC8172758; doi:10.1007/s40201-021-00611-2)
Supplement: Supplementary file 1 — (DOCX 14.5 KB) [file 40201_2021_611_MOESM1_ESM.docx]

Table S1. Operating conditions for LA-ICP-MS system

| *Laser ablation* |  |
| --- | --- |
| Instrument | CETAC LSX-500, Nd-YAG |
| Wavelength (nm) | 266 |
| Pulse duration (ns) | 5 |
| Ablation frequency (Hz) | 10 |
| Spot size (µm) | 50 |
| Laser energy (mJ) | 5,4 |
| Scan rate (µm s^-1^) | 80 |
| Scan method | Mapping 2D |
| *ICP-MS* |  |
| Instrument | PE Sciex ELAN 6100 DRC II |
| Nebulizer gas flow (L min^-1^) | 1.0 |
| Auxilary gas flow (L min^-1^) | 1.2 |
| Plasma gas flow (L min^-1^) | 16 |
| RF power (W) | 1350 |
| Detector | Dual (pulse counting and analog mode) |
| Measured mass | ^26^Mg, ^43^Ca, ^55^Mn, ^59^Co, ^63^Ni, ^65^Cu, ^66^Zn, ^88^Sr, ^111^Cd, ^208^Pb |
| Monitored mass | ^13^C, ^31^P |
